# Nanophthalmos presenting with acute refractory angle-closure glaucoma: acute-phase histopathological evidence and bilateral comparative findings

**DOI:** 10.1186/s12886-026-05087-y

**Published:** 2026-06-30

**Authors:** Chunming Chen, Min Li, Zehao Liu, Chengyao Qin, Wenxiu Sun, Shujun Zhu, Jieqing Cai

**Affiliations:** Liyang Hospital of Chinese Medicine, Liyang, China

**Keywords:** Nanophthalmos, Angle-closure glaucoma, Sclera, Histopathology, Sclerostomy, Acute glaucoma

## Abstract

**Background:**

Nanophthalmos is a rare congenital ocular disorder characterized by a microphthalmic eye with abnormally thickened sclera. Although scleral histopathology has been described in elective surgical cases with normal intraocular pressure (IOP), scleral architecture during acute IOP elevation exceeding 60 mmHg remains unclear. This article reports unique histopathological findings obtained during an episode of refractory acute angle-closure glaucoma.

**Case presentation:**

A 63-year-old man with lifelong high hyperopia (corrected with + 10.25 D spectacles) presented with acute severe ocular pain, headache, nausea, and blurred vision in the right eye for 2 days. Best-corrected visual acuity was 20/2000 in the right eye and 20/70 in the left eye; IOP was > 60 mmHg and 18 mmHg, respectively. Examination revealed extremely shallow anterior chamber (2.15 mm), corneal edema, and nuclear cataract in the right eye; the fellow eye showed similar anterior chamber depth (2.23 mm) with slit-like angle opening, remaining in a preclinical state as an internal control. Axial lengths were 16.3 mm and 16.5 mm, with markedly thickened sclera in both eyes on B-scan ultrasonography. Despite maximal medical therapy, IOP remained > 60 mmHg. Combined lamellar sclerectomy with full-thickness sclerostomy, phacoemulsification with intraocular lens implantation, and posterior capsulotomy was performed, with scleral tissue obtained for histopathological examination. On the first postoperative day, IOP decreased to 17 mmHg, with inflammation resolving within 1 week and scattered superficial retinal hemorrhages completely resolving within 6 weeks. At 8-month follow-up, best-corrected visual acuity improved to 20/80 (with spectacles correcting residual hyperopia of + 10.5 D) with IOP stabilized at 15–18 mmHg without medication. The left eye remained stable (visual acuity 20/70, IOP 16–19 mmHg). Histopathological analysis revealed disorganized collagen architecture in nanophthalmic sclera, showing irregular fiber bundles and loosely arranged connective tissue; in contrast, normal control sclera showed dense, regularly layered collagen with uniform tissue structure.

**Conclusions:**

This case provides the first acute-phase histopathological evidence that characteristic scleral abnormalities in nanophthalmos persist during IOP elevation >60 mmHg, supporting their congenital etiology. Combined sclerostomy and cataract surgery successfully treated refractory nanophthalmos-associated glaucoma in the acute setting, and bilateral findings provide valuable comparative data supporting prophylactic surgical intervention in the fellow eye.

## Background

Nanophthalmos is a rare congenital ocular disorder characterized by a microphthalmic eye with normal internal anatomy, typically presenting with axial length less than 20 mm and high hyperopia ranging from + 8.00 D to + 25.00 D [1, 2], with an estimated prevalence of 1-1.5 per 10,000 [3]. This condition is associated with significantly increased risk of sight-threatening complications including secondary angle-closure glaucoma, uveal effusion syndrome, and malignant glaucoma [4–6].

The pathophysiological core of nanophthalmos is abnormal scleral architecture. Histopathological studies have demonstrated markedly thickened sclera with disorganized collagen fiber arrangement, altered glycosaminoglycan composition, and reduced elasticity [7–14]. Trelstad et al. [7] first described irregularly arranged, thickened collagen fibers with serrated edges in nanophthalmic sclera, and subsequent studies confirmed these findings and identified increased fibronectin expression, suggesting abnormal extracellular matrix composition [9].

Nanophthalmos-associated glaucoma poses substantial surgical challenges due to the high risks of uveal effusion syndrome and malignant glaucoma [4, 15, 16]. Scleral sclerostomy reduces these complications in elective cases with preoperative IOP ranging from 14 to 30 mmHg [17], but evidence for its efficacy in acute refractory cases with IOP exceeding 60 mmHg remains limited. Importantly, all previous histopathological studies were conducted on scleral specimens obtained during elective surgery with relatively normal IOP [7–10]. Scleral architecture during acute IOP elevation, such as during an acute angle-closure attack with medically refractory IOP > 60 mmHg, remains unclear. This knowledge gap has important clinical significance as it affects surgical decision-making, particularly regarding the timing of intervention and the rationale for prophylactic surgery in the fellow eye.

This case report describes a patient with nanophthalmos who presented with unilateral acute refractory angle-closure glaucoma, providing a unique opportunity to examine scleral histopathology during acute IOP elevation (> 60 mmHg) and anatomical comparative data from the preclinical fellow eye.

## Case presentation

**Fig. 1:**
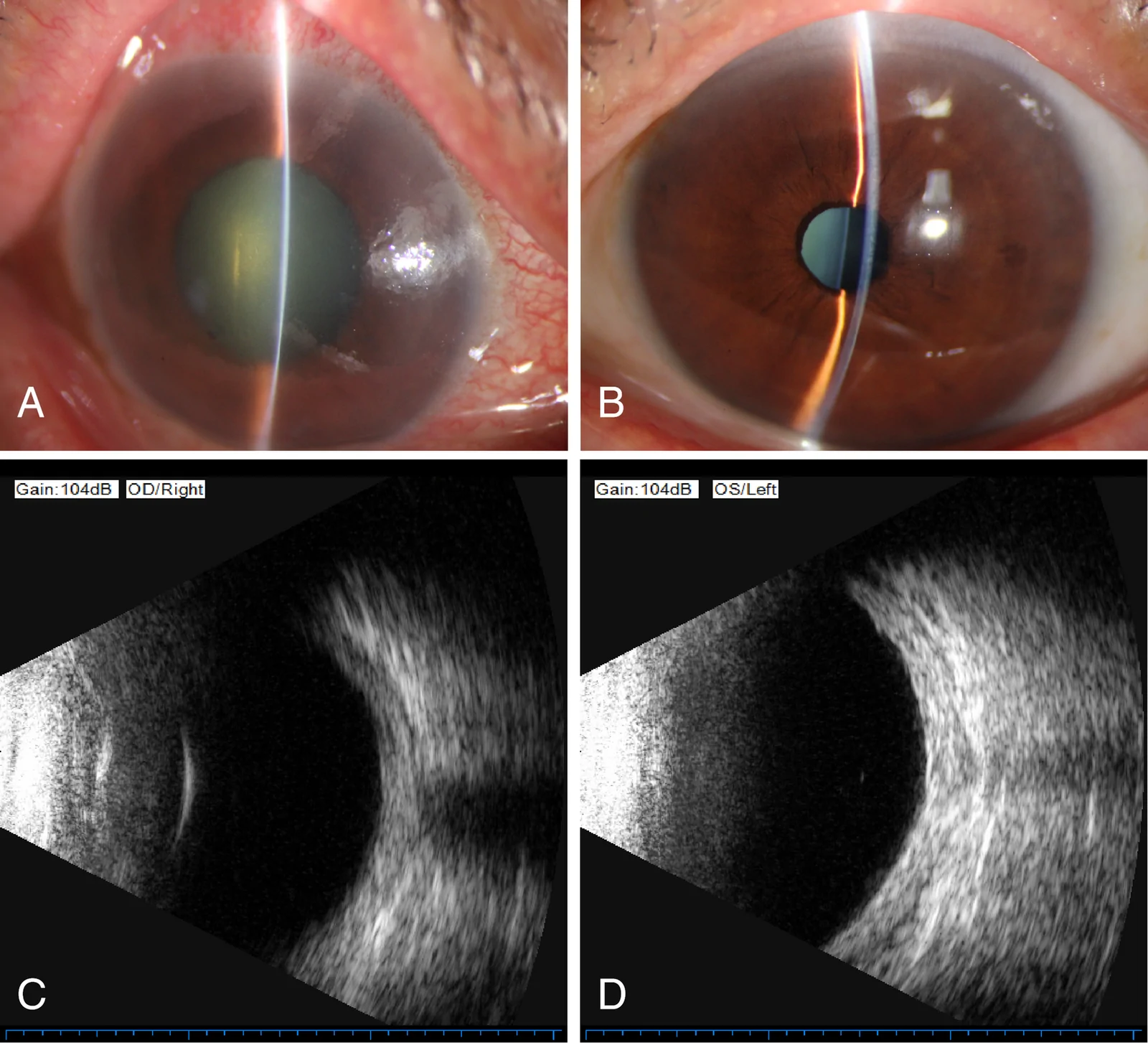
Bilateral anterior segment findings and scleral thickness in nanophthalmos. **A** Slit-lamp photograph of the right eye with optical section demonstrating markedly shallow anterior chamber (depth 2.15 mm). **B** Slit-lamp photograph of the left eye showing similarly shallow anterior chamber (depth 2.23 mm) in the preclinical fellow eye. **C** B-scan ultrasonography of the right eye revealing markedly thickened sclera (qualitative assessment) during acute IOP elevation. **D** B-scan of the fellow eye showing comparable scleral thickening despite normal IOP, suggesting congenital structural abnormalities independent of pressure status

A 63-year-old man with lifelong high hyperopia (corrected with + 10.25 D spectacles) presented with acute severe ocular pain, headache, nausea, and blurred vision in the right eye for 2 days. He denied any history of glaucoma, ocular surgery, or family history of glaucoma. Examination revealed best-corrected visual acuity of 20/2000 in the right eye and 20/70 in the left eye; IOP was > 60 mmHg in the right eye and 18 mmHg in the left eye. Corneal diameters (horizontal white-to-white) were 10.4 mm OD and 10.6 mm OS. Keratometry showed K1 44.12 D and K2 45.20 D OD, K1 43.38 D and K2 45.00 D OS. Lens thickness was 4.82 mm OD and 4.74 mm OS. Optic disc examination revealed cup-to-disc ratios of 0.3 OD and 0.3 OS. Slit-lamp examination of the right eye showed extremely shallow anterior chamber (2.15 mm), corneal edema, and nuclear cataract (Fig. 1A), with anterior segment optical coherence tomography confirming completely closed angles; the left eye showed similar shallow anterior chamber (2.23 mm) with clear cornea and early nuclear cataract (Fig. 1B), with slit-like angle opening. B-scan ultrasonography demonstrated markedly thickened sclera in both eyes (Fig. 1C and D), with axial lengths of 16.3 mm and 16.5 mm, respectively. The left eye served as an internal control, demonstrating nearly identical anatomical features but remaining in a preclinical state with normal IOP and open angles, providing a rare opportunity for comparative pathological analysis.

Gonioscopic examination was precluded by corneal edema in the right eye and poor visualization in the left eye. Anterior segment optical coherence tomography (AS-OCT) confirmed complete angle closure in the right eye with iris apposition to the corneal endothelium. In the left eye, AS-OCT revealed slit-like angle opening; however, the mid-peripheral iris was apposed to the corneal endothelium, indicating a critically narrow angle at high risk of closure.

**Fig. 2:**
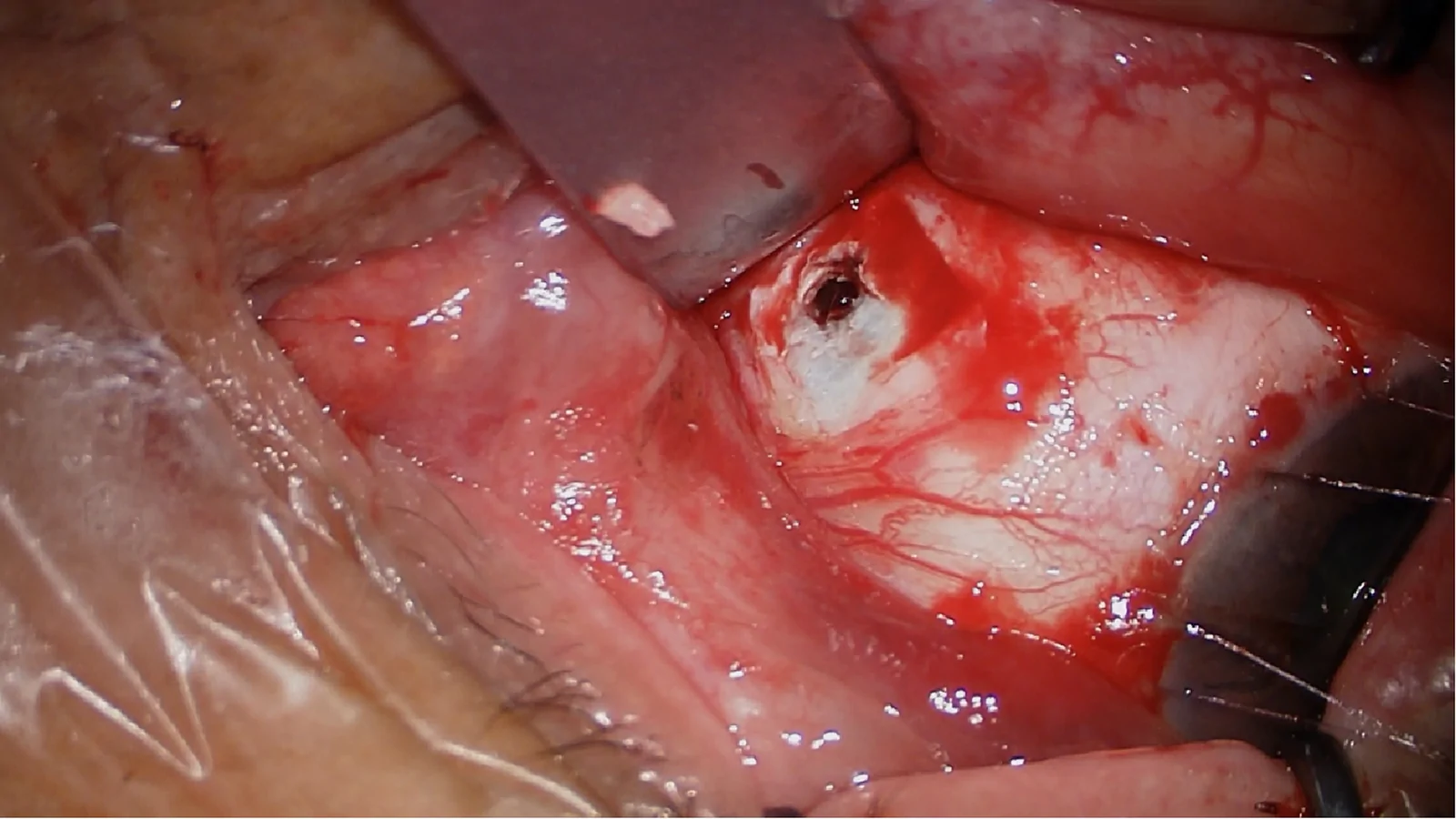
Intraoperative view of sclerostomy. Intraoperative photograph demonstrating nasal scleral sclerostomy (arrow) prior to cataract extraction. Combined surgery included 4×4 mm lamellar sclerectomy with 1×1 mm full-thickness sclerostomy at both nasal and temporal quadrants

Despite emergency peripheral iridotomy and maximal medical therapy including intravenous mannitol, topical hypotensive agents, and oral acetazolamide, IOP in the right eye remained > 60 mmHg. Given the refractory nature of IOP elevation > 60 mmHg despite maximal medical therapy for 48 h, the surgical decision was to perform lamellar sclerectomy with full-thickness sclerostomy at the equatorial region to address the underlying uveal effusion risk and nanophthalmic anatomy, rather than conventional trabeculectomy. Under local anesthesia, 4 × 4 mm lamellar sclerectomy with 1 × 1 mm full-thickness sclerostomy was performed at the equatorial region (10–12 mm posterior to the limbus) at nasal and temporal quadrants (Fig. 2). Intraoperative precautions included gradual decompression during sclerostomy creation to prevent sudden IOP drop and subsequent uveal effusion or choroidal hemorrhage, preservation of at least one-half of the scleral thickness in the lamellar bed to maintain structural integrity, and sequential performance of sclerostomy before cataract extraction to allow early decompression. Notably, after sclerostomy creation, IOP remained elevated; therefore, slow vitreous cavity fluid aspiration through the pars plana was performed to further reduce IOP prior to cataract extraction. Subsequently, phacoemulsification with intraocular lens implantation (+ 30.0 D; IOL power calculated using Barrett Universal II formula was + 50.0 D with target refraction + 2.00 D, but the maximum commercially available IOL power was + 30.0 D), limited anterior vitrectomy, and posterior capsulotomy were performed through a 2.8 mm clear corneal incision, with excised scleral tissue obtained for histopathological analysis.

On the first postoperative day, IOP decreased to 17 mmHg; anterior chamber inflammation resolved within 1 week with topical corticosteroids; scattered superficial retinal hemorrhages completely resolved within 6 weeks; and no complications of uveal effusion, choroidal detachment, or malignant glaucoma occurred. At 8-month follow-up, best-corrected visual acuity improved to 20/80 (with spectacles correcting residual hyperopia of + 10.5 D) with IOP stabilized at 15–18 mmHg without antiglaucoma medication. The left eye remained stable (visual acuity 20/70, IOP 16–19 mmHg) without evidence of angle closure or glaucoma progression; the patient declined prophylactic surgery for the left eye and continues under observation.

## Histopathological findings

**Fig. 3:**
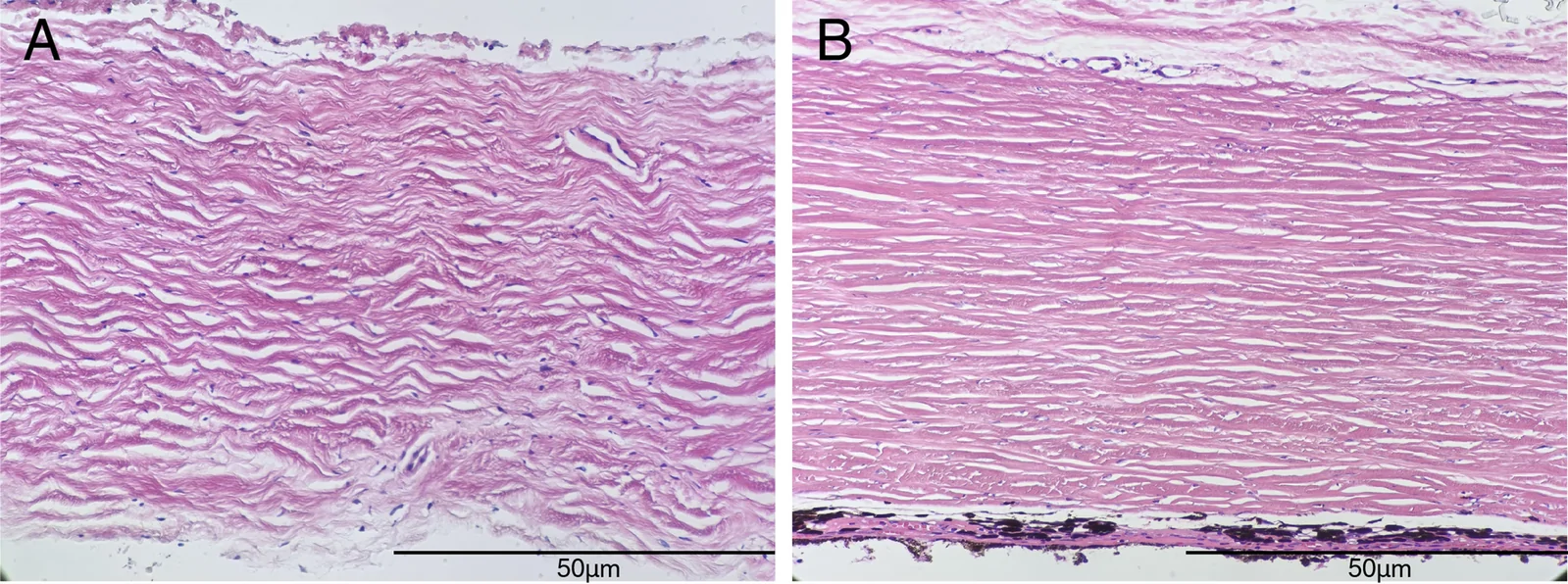
Histopathological findings. **A** Nanophthalmic sclera (H&E, ×200) obtained during acute-phase surgery showing collagen hyperplasia with disorganized fiber architecture and loose connective tissue arrangement. Scale bar = 50 μm. **B** Normal control sclera (H&E, ×200) from a 65-year-old patient (axial length 24.2 mm, no ocular disease) showing dense, regularly arranged collagen lamellae. Scale bar = 50 μm

Scleral tissue obtained from the right eye during surgery was fixed in 10% neutral buffered formalin, embedded in paraffin, and sectioned at 5 μm thickness, with hematoxylin and eosin (H&E) staining performed for routine histopathological examination. Histopathological analysis of the excised nanophthalmic sclera revealed markedly abnormal collagen architecture: H&E staining at 200× magnification showed disorganized collagen bundles with irregular fibers and loosely arranged connective tissue (Fig. 3A), with collagen fibers exhibiting a twisted and haphazard interweaving pattern, lacking the regular lamellar arrangement seen in normal sclera. In contrast, normal control sclera obtained from a 65-year-old patient (axial length 24.2 mm, no ocular disease) at the same anatomical position showed dense, regularly arranged collagen lamellae with uniform tissue structure and parallel fiber arrangement (Fig. 3B).

## Discussion and conclusion

This case report presents unique acute-phase histopathological findings from a nanophthalmic eye during medically refractory angle-closure glaucoma attack (IOP > 60 mmHg), with anatomical comparative data from the preclinical fellow eye. Comparison with previous histopathological studies reveals both consistent and novel findings (Table 1). Previous studies by Trelstad et al. [7], Uyama et al. [8], Yue et al. [9], and Rajendrababu et al. [10] described irregularly arranged, thickened collagen fibers with disorganized architecture in nanophthalmic sclera, and our findings are consistent with these reports, confirming that characteristic scleral abnormalities persist even during acute IOP elevation > 60 mmHg.

**Table 1:** Comparison of scleral histopathological findings in nanophthalmos

Study	Year	Number of cases	IOP at surgery	Key findings
Trelstad et al. [7]	1982	2 cases	Normal range (during uveal effusion episode)	Disorganized collagen fibers, proteoglycan accumulation, scleral thickening
Uyama et al. [8]	2000	19 eyes (nanophthalmic type 6, abnormal scleral type 11, normal scleral type 2)	Normal range	Nanophthalmic and abnormal scleral types: disorganized collagen fiber bundles, proteoglycan deposition; lamellar sclerectomy was effective
Yue et al. [9]	1986	1 case	Normal range (during uveal effusion episode)	Irregularly arranged collagen fibers, increased fibronectin, abnormal glycosaminoglycan metabolism
Rajendrababu et al. [10]	2022	4 cases (4 eyes)	14–30 mmHg	Disorganized collagen fibers, strongly positive fibronectin expression; more severe disorganization in eyes with AL < 17 mm
Current case	2025	1 case	Acute phase > 60 mmHg	Disorganized collagen architecture, loose connective tissue arrangement

Histopathological comparison between nanophthalmic sclera and normal control tissue obtained from a patient without nanophthalmos highlighted marked structural differences: normal sclera demonstrated dense, regularly arranged collagen lamellae typical of healthy eyes, while nanophthalmic sclera showed disorganized fiber architecture with loose connective tissue arrangement. This contrast underscores the intrinsic structural abnormalities in nanophthalmos rather than variations within the normal range.

The key distinction of our case is the timing of specimen acquisition and the anatomical comparative data. All previous histopathological studies were conducted on scleral specimens obtained during elective surgery with preoperative IOP ranging from 14 to 30 mmHg [7–10]. In contrast, our specimen was obtained during emergency surgery for acute refractory angle-closure glaucoma with IOP > 60 mmHg despite maximal medical therapy for 48 h. This unique circumstance provides important insights into the nature of scleral abnormalities in nanophthalmos. The persistence of characteristic scleral abnormalities during acute IOP elevation supports their congenital nature; if collagen disorganization and loose connective tissue arrangement were secondary to chronic or acute IOP elevation, one might expect more severe changes or different patterns in an acute case. However, the histopathological features were consistent with previous reports from eyes without acute IOP elevation, suggesting these are intrinsic, developmental abnormalities rather than acquired changes.

This interpretation is strongly supported by the bilateral nature of this case: the fellow eye demonstrated nearly identical anatomical features, including comparable axial length (16.5 mm vs. 16.3 mm), anterior chamber depth (2.23 mm vs. 2.15 mm), and scleral thickness on B-scan ultrasonography, yet remained in a preclinical state with normal IOP (18 mmHg) and open angles. The presence of similar structural abnormalities in the fellow eye with normal IOP provides compelling evidence that these changes are congenital rather than pressure-induced. The loose connective tissue arrangement observed in our case likely increases resistance to fluid and macromolecular transport through the sclera, explaining the susceptibility to uveal effusion syndrome in nanophthalmos [8]. Despite acute IOP elevation > 60 mmHg, these characteristic structural abnormalities persisted, further supporting their congenital developmental origin.

It is important to note that histopathological examination was performed only on the right eye scleral specimen obtained during surgery. No tissue was obtained from the left eye, which remains under observation. The comparison is therefore based on anatomical and imaging features rather than bilateral histopathology.

From a clinical perspective, this case demonstrates that combined sclerostomy and cataract surgery successfully treated nanophthalmos-associated glaucoma with medically refractory IOP elevation > 60 mmHg. Prior randomized trials have demonstrated the efficacy of prophylactic sclerostomy in elective cases with preoperative IOP ranging from 14 to 30 mmHg [17], and our case extends those findings to acute decompensation, confirming the rationale for prophylactic scleral decompression even in emergent settings. The surgical technique of lamellar sclerectomy with full-thickness sclerostomy creates an outflow pathway for fluid while avoiding complications associated with more extensive scleral resection; 4 × 4 mm lamellar sclerectomy with 1 × 1 mm full-thickness sclerostomy at two quadrants (nasal and temporal) provides adequate decompression while maintaining scleral structural integrity. The postoperative course in our case was favorable, with IOP control achieved without antiglaucoma medication at 8 months and no complications of uveal effusion, choroidal detachment, or malignant glaucoma.

Management of the fellow eye in nanophthalmos remains controversial: some authors advocate prophylactic surgery given the high risk of complications [17], while others suggest careful observation with laser peripheral iridotomy [4, 18]. In our case, despite counseling about the high risk of acute angle-closure attack, the patient declined prophylactic surgery for the left eye. The histopathological findings from the right eye and bilateral anatomical similarities support the rationale for prophylactic intervention, which may be discussed again with the patient during follow-up visits.

This case has several limitations: first, it is a single case report, and findings may not be generalizable; second, we were unable to obtain scleral tissue from the fellow eye for direct histopathological comparison due to the patient’s decision to defer surgery; third, quantitative analysis of collagen fiber density, thickness, and arrangement was not performed; fourth, only H&E staining was performed due to limited tissue volume from the small sclerostomy specimen, precluding additional specialized staining such as Masson’s trichrome or immunohistochemistry; fifth, long-term follow-up data beyond 8 months are not yet available. Future studies with larger sample sizes, quantitative histopathological analysis, and longer follow-up would strengthen these findings.

In conclusion, this case provides the first acute-phase histopathological evidence that characteristic scleral abnormalities in nanophthalmos persist during IOP elevation > 60 mmHg, supporting their congenital nature. Combined sclerostomy and cataract surgery successfully treated refractory nanophthalmos-associated glaucoma in the acute setting, and bilateral findings provide valuable comparative data supporting prophylactic surgical intervention in the fellow eye.

## Data Availability

All data generated or analyzed during this study are included in this published article and are available from the corresponding author on reasonable request.
